# The Selective SGLT2 Inhibitor Ipragliflozin Has a Therapeutic Effect on Nonalcoholic Steatohepatitis in Mice

**DOI:** 10.1371/journal.pone.0146337

**Published:** 2016-01-05

**Authors:** Yasushi Honda, Kento Imajo, Takayuki Kato, Takaomi Kessoku, Yuji Ogawa, Wataru Tomeno, Shingo Kato, Hironori Mawatari, Koji Fujita, Masato Yoneda, Satoru Saito, Atsushi Nakajima

**Affiliations:** Department of Gastroenterology and Hepatology, Yokohama City University Graduate School of Medicine, Yokohama, Japan; University of Basque Country, SPAIN

## Abstract

**Background & Aims:**

In recent years, nonalcoholic steatohepatitis (NASH) has become a considerable healthcare burden worldwide. Pathogenesis of NASH is associated with type 2 diabetes mellitus (T2DM) and insulin resistance. However, a specific drug to treat NASH is lacking. We investigated the effect of the selective sodium glucose cotransporter 2 inhibitor (SGLT2I) ipragliflozin on NASH in mice.

**Methods:**

We used the Amylin liver NASH model (AMLN), which is a diet-induced model of NASH that results in obesity and T2DM. AMLN mice were fed an AMLN diet for 20 weeks. SGLT2I mice were fed an AMLN diet for 12 weeks and an AMLN diet with 40 mg ipragliflozin/kg for 8 weeks.

**Results:**

AMLN mice showed steatosis, inflammation, and fibrosis in the liver as well as obesity and insulin resistance, features that are recognized in human NASH. Ipragliflozin improved insulin resistance and liver injury. Ipragliflozin decreased serum levels of free fatty acids, hepatic lipid content, the number of apoptotic cells, and areas of fibrosis; it also increased lipid outflow from the liver.

**Conclusions:**

Ipragliflozin improved the pathogenesis of NASH by reducing insulin resistance and lipotoxicity in NASH-model mice. Our results suggest that ipragliflozin has a therapeutic effect on NASH with T2DM.

## Introduction

In recent decades, the metabolic syndrome has become increasingly prevalent and the incidence of nonalcoholic fatty liver disease (NAFLD) has also increased [1–3]. NAFLD is associated with the metabolic syndrome [4–7]. NAFLD is a major form of chronic liver disease not associated with significant consumption of alcohol. NAFLD is a clinical and pathologic term describing a disease spectrum ranging from nonalcoholic fatty liver (NAFL) to nonalcoholic steatohepatitis (NASH), cirrhosis, and hepatocellular carcinoma [8]. Obesity and type 2 diabetes mellitus (T2DM) are important risk factors for NAFLD. In the obese population, the prevalence of NAFLD is 4.6-times higher than that in normal individuals [9]. Among individuals with DM, 33–50% of patients have NAFLD [10]. The common feature between T2DM and NAFLD is insulin resistance [11–13]. The insulin-sensitizing agent pioglitazone has a beneficial effect upon NASH, but its efficacy and safety in long-term studies have not been confirmed [14]. Effective drug therapy for NASH has not been established.

Sodium glucose cotransporter 2 inhibitors (SGLT2Is) have been developed for T2DM treatment. SGLT2Is prevent the reabsorption of glucose filtered by glomeruli and increase urinary excretion of glucose [15–17]. Ipragliflozin is a selective inhibitor of SGLT2 that is orally administered. Ipragliflozin decreases blood levels of glucose and hemoglobin (Hb)_A1c_ and improves insulin resistance in T2DM patients and in several models of DM [18–23]. In addition, ipragliflozin has been shown to improve dyslipidemia and liver steatosis in streptozotocin–nicotinamide-induced T2DM mice fed a high-fat diet [21]. These mice were obese and had insulin resistance and fatty liver, but did not have steatohepatitis or fibrosis. Hayashizaki-Someya et al. [24] reported that ipragliflozin has a prophylactic effect on hepatic fibrosis in rats fed a choline-deficient L-amino acid-defined (CDAA) diet. Mice fed a CDAA diet are used frequently as a nutritional model of NASH. A CDAA diet induces an elevation in aminotransferase levels and histologic changes characterized by steatosis, inflammation, hepatocyte necrosis, and tissue fibrosis [25]. However, this model does not express obesity and insulin resistance, which are features of NASH in humans. When investigating if ipragliflozin has a therapeutic effect upon NASH, the model employed must be similar to NASH seen in humans.

We investigated the effect of ipragliflozin upon NASH in the Amylin liver NASH model (AMLN). AMLN is a dietary model of NASH that expresses obesity, insulin resistance, and the three stages of NAFLD (steatosis, steatohepatitis with fibrosis, and cirrhosis) [26].

## Materials and Methods

### Drugs and diets

Ipragliflozin L-proline was obtained from Astellas Pharma (Ibaraki, Japan). A basal diet (BD) was prepared containing 22% protein, 6% fat, and 47% carbohydrate. A diet enriched in fat (40% kcal, Primex partially hydrogenated vegetable oil shortening), fructose (22% by weight), and cholesterol (2% by weight) (catalog number D09100301; Research Diets, New Brunswick, NJ, USA) was purchased. Clapper et al. [27] reported that this diet (the AMLN diet) induces all stages of NAFLD for periods >20 weeks in C57BL/6J mice. The diet D09100301 with 40 mg ipragliflozin L-proline/kg of diet (D14101901; Research Diets) was purchased.

### Animal experiments

This study was in accordance with the Guidelines for the Care and Use of Laboratory Animals set by Yokohama City University Medical School (Yokohama, Japan). The protocol was approved by the Committee on the Ethics of Animal Experiments of the University of Yokohama City University Medical School (Permit Number: F-A-15-016). All surgery was performed with sodium pentobarbital anesthesia, and all efforts were made to minimize suffering. The experimental protocol is outlined in S1 Fig.

Six-week-old male C57BL/6J mice were obtained from CLEA Japan (Tokyo, Japan). After a 2-week acclimatization period, the groups of mice were fed as follows: BD mice fed a BD for 20 weeks, AMLN mice fed D09100301 for 20 weeks, and SGLT2I mice fed D09100301 for 12 weeks followed by D14101901 for 8 weeks. All animals were housed under conventional conditions with controlled temperature, humidity, and light (12-h light–dark cycle) and provided with food and water.

### Biochemical analyses

Serum levels of aspartate aminotransferase (AST), alanine aminotransferase (ALT), total cholesterol, fasting plasma glucose, insulin, and free fatty acids (FFAs) were measured by a local laboratory (SRL, Tokyo, Japan). As an alternative method for assessment of insulin resistance, the homeostasis model assessment of insulin resistance (HOMA-IR) was calculated using the following formula:

HOMA-IR = insulin (μU/mL) × fasting plasma glucose (mg/dL)/405.

Levels of triglycerides (TGs) and FFAs in total lipid extracts of the liver were determined by colorimetric assays (Wako Pure Chemical Industries, Osaka, Japan).

### Histological and immunohistochemical analyses

Paraffin-embedded sections were stained with hematoxylin and eosin (H&E), Masson’s trichrome (MT) or Sirius red (SR). For lipid staining, frozen sections were stained with oil red O and counterstained with hematoxylin. The NAFLD activity score and fibrosis stage were scored by an outsourcing company (Allisere, Tokyo, Japan) in a blind manner according to the method of Kleiner et al. (S1 Table) [27]. Immunohistochemistry was carried out on cryostat liver sections (thickness, 7 μm). Sections were incubated with primary antibodies and stained with Alexa Fluor-conjugated secondary antibodies (Cell Signaling Technology, Danvers, MA, USA). Apoptosis was assessed by TUNEL staining of paraffin-embedded slides. Images of five random fields of each section were selected and the number of apoptotic cells per field was counted. To quantify the area of SR staining and α-smooth muscle actin (α-SMA) staining, images of five random fields of each section were processed with Photoshop Elements v13 (Adobe Systems, San Jose, CA, USA). Each value was expressed as the percentage of the total area of the section.

### RNA isolation and real-time polymerase chain reaction (PCR) analyses

Total RNA was extracted from samples of liver tissue using an RNeasy mini kit (Qiagen, Tokyo, Japan). Messenger RNA (mRNA) of murine α-SMA, collagen 1α1, acetyl-CoA carboxylase (ACC), sterol regulatory element-binding protein 1c (SREBP1c), peroxisome proliferator activated receptor α (PPARα), carnitine palmitoyltransferase 1A (CPT1A), microsomal triglyceride transfer protein (MTTP), and β-actin were determined in liver tissue using a fluorescence-based reverse transcription-PCR and an ABI PRISM 7700 Sequence Detection System (Life Technologies, Carlsbad, CA, USA). SREBP1c and ACC1 are key factors of lipid synthesis [28]. PPARα is a key factor for hepatic lipid metabolism because it stimulates the transcription of PPARα-regulated genes [29, 30]. CPT-1 is a key regulatory enzyme of β-oxidation and is required for the transport of long-chain fatty acids into mitochondria [31]. MTTP has a pivotal role in export of very-low-density lipoprotein from the liver [32].

### Statistical analyses

Data were recorded as mean ± standard deviation (SD). Differences between the two groups were assessed using the Student’s t-test and p<0.05 was considered significant. Statistical analyses were carried out using JMP v11.2.0 (SAS Institute, Cary, NC, USA).

## Results

### Effect of ipragliflozin on physical, biochemical, and pathological parameters in model mice

Table 1 shows the characteristics of the model mice. There were no differences in daily food intake among the three groups. AMLN mice exhibited obesity, insulin resistance, and liver injury in comparison with BD mice. No difference in body weight and weight of epididymal adipose tissue between AMLN mice and SGLT2I mice was observed, but liver weight was significantly lower in SGLT2I mice compared with AMLN mice. SGLT2I mice showed lower levels of total cholesterol than AMLN mice. Ipragliflozin reduced the levels of fasting plasma glucose and insulin, and improved insulin resistance significantly in SGLT2I mice. Levels of aminotransferase and FFAs were decreased significantly in SGLT2I mice. Only one AMLN mouse showed hepatocyte ballooning, but AMLN mice were affected by steatosis, inflammation, and fibrosis in the liver according to pathological evaluations (Table 2).

**Table 1:** Characteristics of model mice.

Parameters	BD	AMLN	SGLT2I
Food intake (g/day)	3.41±0.13	3.46±0.13	3.58±0.21
Body weight (g)	28.9±1.4	39.7±2.8*	39.6±1.1*
Liver weight (g)	1.26±0.10	3.32±0.37*	2.77±0.42*†
Weight of epididymal adipose tissue (g)	0.23±0.08	1.09±0.18*	1.10±0.10*
Fasting plasma glucose (mg/dL)	90.7±32.2	203.5±55.2*	142.4±41.6*†
Insulin (ng/mL)	0.32±0.26	1.13±0.56*	0.48±0.26†
HOMA-IR	1.96±2.0	15.5±9.7*	4.8±3.3†
Total cholesterol (mg/dL)	97.7±5.4	270.1±47.1*	200.2±23.8*†
AST (IU/L)	40.5±8.1	400.4±136.1*	194.0±114.6*†
ALT (IU/L)	26.5±15.0	471.9±149.0*	178.8±122.2*†
Free fatty acid (uEQ/L)	938±293.1	1325.4±140.8*	1154.2±106.9†

HOMA-IR, the homeostasis model assessment of insulin resistance; AST, aspartate aminotransferase; ALT, alanine aminotransferase. Data are the mean ± SD (n = 5–8). Significance was determined using the Student’s *t*-test:

* p<0.05 *versus* BD mice

^†^ p<0.05 *versus* AMLN mice

**Table 2:** Liver pathology scores.

Parameter	BD	AMLN	SGLT2I
Steatosis	0	3.0*	2.4±0.6*†
Lobular Inflammation	0	1.8±0.8*	0.8±0.8*†
Hepatocyte Ballooning	0	0.2±0.4	0
NAS	0	5.0±0.7*	3.2±0.8*†
Fibrosis stage	0	1.6±0.5*	0.8±0.4*†

NAFLD activity score (NAS) and fibrosis stage were scored according to the method described by Kleiner et al. [27], as outlined in S1 Table. NAS, NAFLD Activity Score. Data are the mean ± SD (n = 5 each). Significance was determined using the Student’s *t*-test:

* p<0.05 *versus* BD mice

^†^ p<0.05 *versus* AMLN mice

### Ipragliflozin improved the pathogenesis of NASH in mice

**Fig 1:**
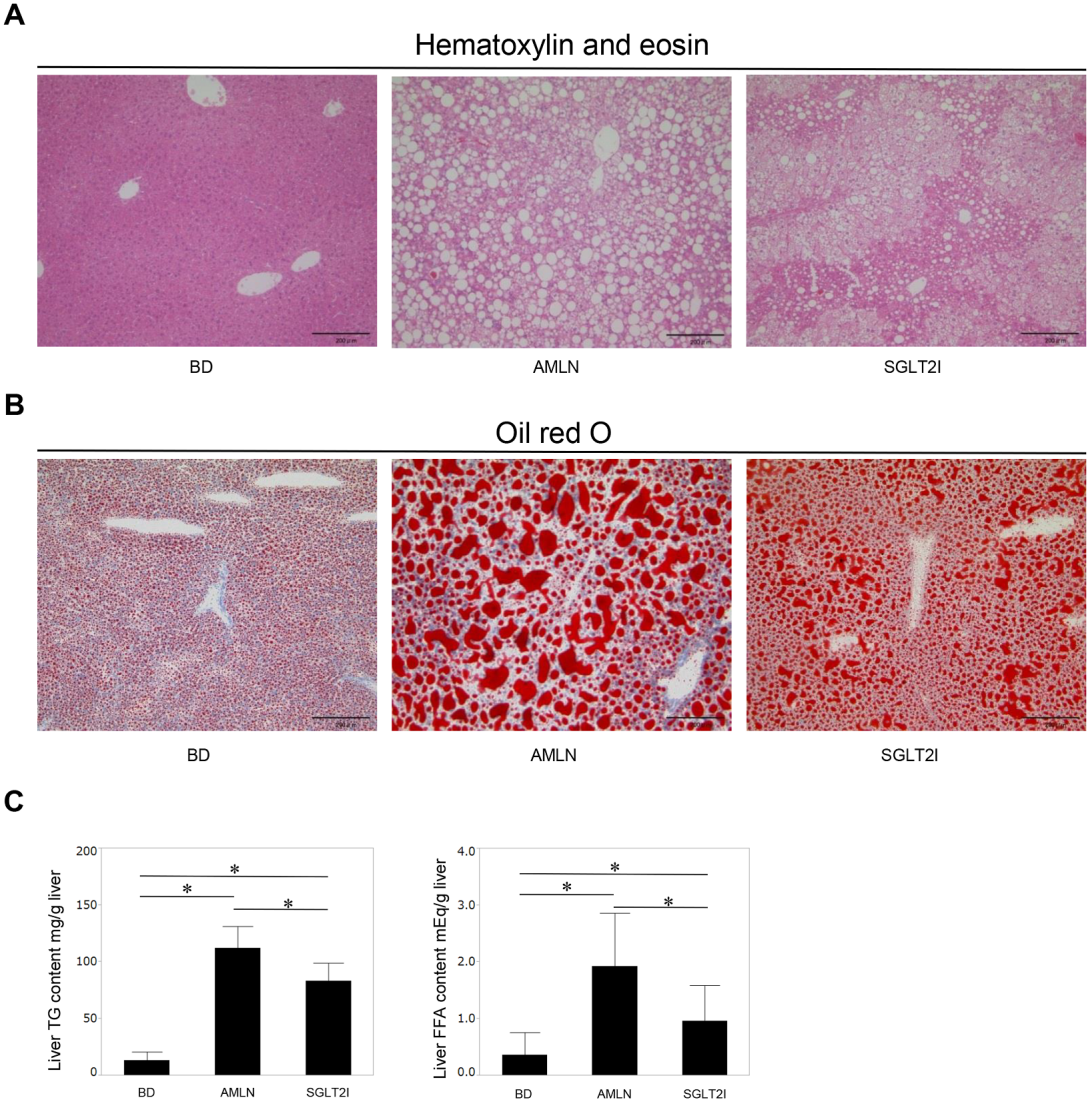
Ipragliflozin improved steatosis and decreased lipid content in the liver. (A) Liver sections from BD mice, AMLN mice and SGLT2I mice. Hematoxylin and eosin (H&E) staining, (B) oil red O staining. Magnification, 200×. Scale bars: 200 μm. (C) Triglyceride (TG) and free fatty acid (FFA) content was measured in the livers of BD, AMLN, and SGLT2I mice (n = 4–6). Results are the mean ± SD. Significance was tested using the Student’s *t*-test (*p<0.05).

**Fig 2:**
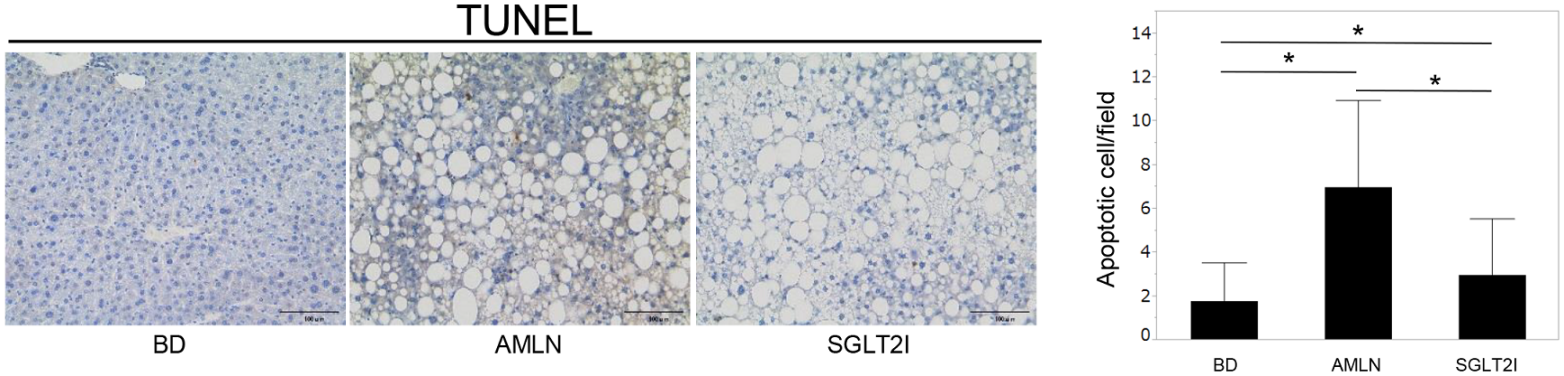
Ipragliflozin improved liver inflammation and suppressed hepatocyte apoptosis. TUNEL-stained paraffin-embedded sections of liver tissue from BD, AMLN, and SGLT2I mice. Magnification, 400×. Scale bars: 100 μm. The number of apoptotic cells per field was counted in BD, AMLN, and SGLT2I mice (n = 5–8). Results are the mean ± SD. Significance was determined using the Student’s *t*-test (*p<0.05).

**Fig 3:**
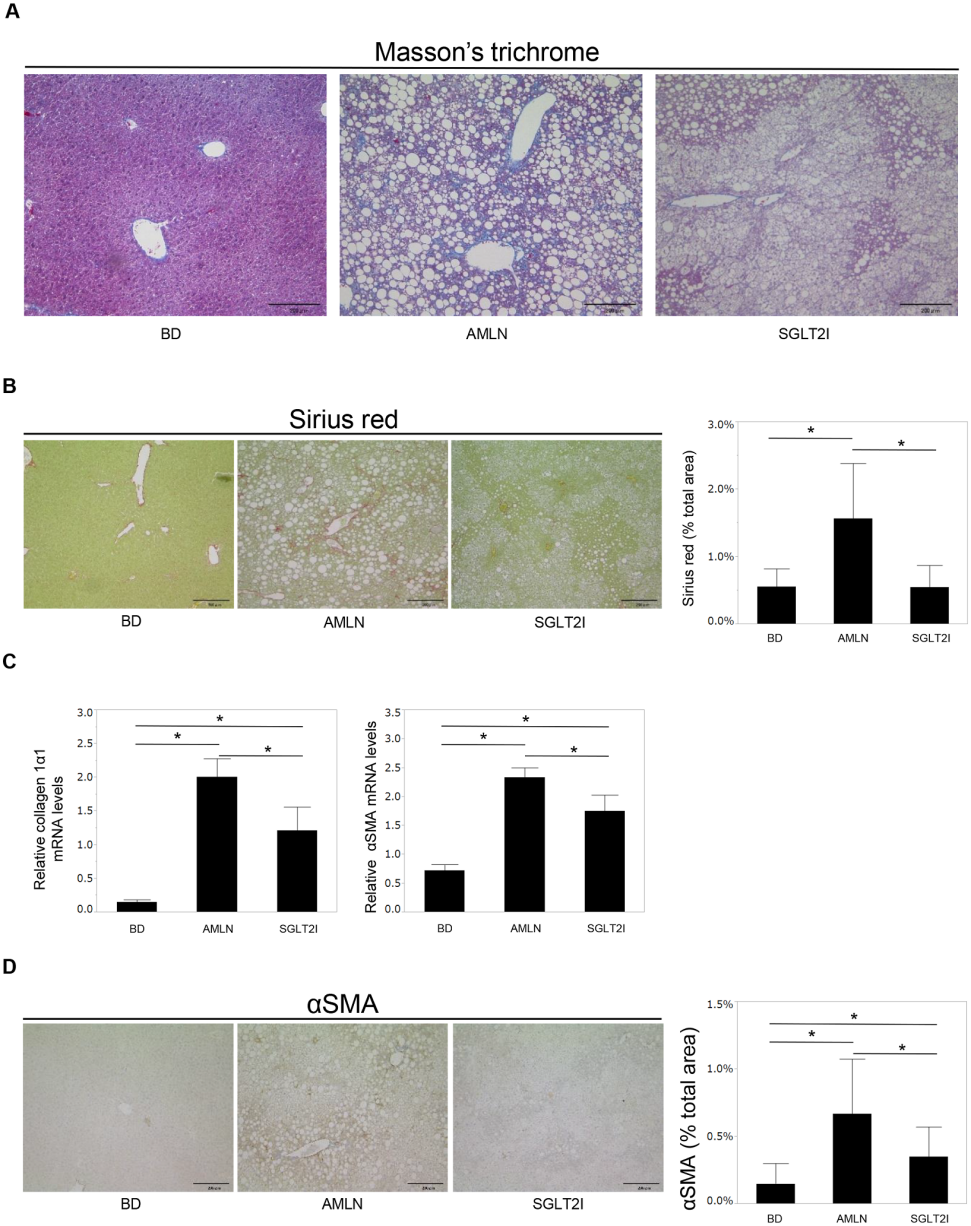
Ipragliflozin improved fibrosis. (A) Liver sections from BD, AMLN and SGLT2I mice. Masson’s trichrome (MT), (B) Sirius red (SR) staining. Areas of SR staining in the liver of BD, AMLN, and SGLT2I mice (n = 5–8). (C) Expression of collagen 1α1 mRNA, and α-smooth muscle actin α (α-SMA) mRNA in BD, AMLN, and SGLT2I mice (n = 5–8). (D) α-SMA-stained paraffin-embedded sections of liver tissue from BD, AMLN, and SGLT2I mice. Areas of α-SMA staining in the livers of BD, AMLN, and SGLT2I mice (n = 5–8). Magnification, 200×. Scale bars: 200 μm. Results are the mean ± SD. Significance was determined using the Student’s *t*-test (*p<0.05).

The steatosis grade was significantly lower in SGLT2I mice than in AMLN mice (Table 2). Staining (H&E, oil red O) showed that the livers of SGLT2I mice had fewer and smaller lipid droplets in the peripheral periportal zone in comparison with AMLN mice (Fig 1A and 1B). Ipragliflozin reduced TG and FFA content in the livers of SGLT2I mice (Fig 1C). Ipragliflozin improved liver injury in SGLT2I mice significantly (Table 1) and also decreased the lobular inflammation grade significantly (Table 2). Hepatocyte apoptosis is a key pathological feature of NASH and is associated with progressive inflammation and fibrosis of the liver [33, 34]. The number of apoptotic cells in SGLT2I mice was fewer than that in AMLN mice (Fig 2). The extent of fibrosis was confirmed by staining (MT, SR, α-SMA) and by mRNA expression of collagen 1α1 and α-SMA. MT staining revealed that AMLN mice developed liver fibrosis (Fig 3A), but the fibrosis stage was significantly lower in SGLT2I mice than in AMLN mice (Table 2). In SGLT2I mice, areas of SR staining were significantly smaller than those in AMLN mice (Fig 3B). Furthermore, mRNA levels of collagen 1α1 and α-SMA in SGLT2I mice were significantly lower than those in AMLN mice (Fig 3C). The α-SMA staining and the areas of α-SMA staining were also smaller in SGLT2I mice than in AMLN mice (Fig 3D).

### Ipragliflozin accelerated lipid outflow from the liver in mice

**Fig 4:**
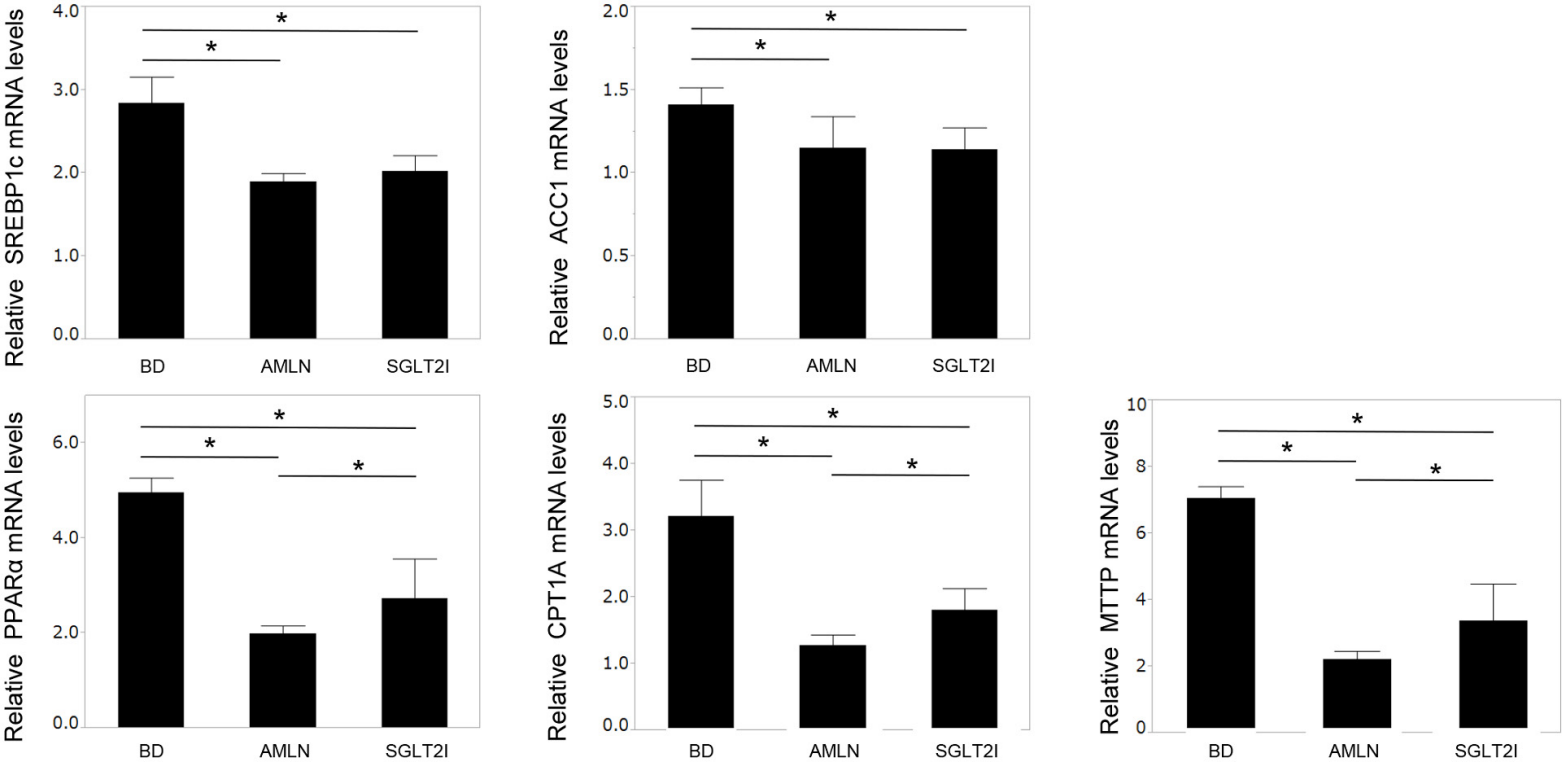
Ipragliflozin accelerated β-oxidation and export of very-low-density lipoprotein. Expression of mRNA of acetyl-CoA carboxylase (ACC), sterol regulatory element-binding protein 1c (SREBP1c), peroxisome proliferator activated receptor α (PPARα), carnitine palmitoyltransferase 1A (CPT1A), and microsomal triglyceride transfer protein (MTTP) in BD, AMLN, and SGLT2I mice (n = 5–8). Results are the mean ± SD. Significance was determined using the Student’s *t*-test (*p<0.05).

We analyzed mRNA expression of ACC1, SREBP1c, PPARα, CPT1A, and MTTP in the liver. The mRNA expressions of ACC1 and SREBP1c are markers of lipid inflow in the liver, while mRNA expressions of PPARα, CPT1A, and MTTP are markers of lipid outflow in the liver. The mRNA expressions of ACC1, SREBP1c, PPARα, CPT1A, and MTTP in AMLN mice and SGLT2I mice were lower than those of BD mice, but mRNA expressions of PPARα, CPT1a, and MTTP in SGLT2I mice were significantly higher than those in AMLN mice (Fig 4).

These results suggested that ipragliflozin had a therapeutic effect on NASH by improving insulin resistance, suppressing hepatocyte apoptosis, and upregulating lipid outflow from the liver.

## Discussion

We investigated the effect of ipragliflozin (selective inhibitor of SGLT2) on NASH in mice. Our results suggested that ipragliflozin had a therapeutic effect on NASH with T2DM.

NAFLD is the most common chronic disease of the liver worldwide. In general, NAFL is non-progressive and benign, whereas NASH can progress to cirrhosis and hepatocellular carcinoma. In terms of pathology, not only liver steatosis but also inflammation and/or fibrosis of the liver are recognized in NASH. In the present study, we used AMLN, which is a diet-induced model of NASH. AMLN induces NASH without reliance on genetic mutations, use of toxins, or nutrient deficiency. An AMLN diet induces all pathological stages of NAFLD for any period >20 weeks in C57BL/6J mice. AMLN also exhibit obesity and insulin resistance [26].

In the present study, AMLN mice showed steatosis, inflammation, and fibrosis of the liver, as well as obesity and insulin resistance (Tables 1 and 2). Thus, AMLN has similar biochemical and pathological characteristics to that of human NASH. These characteristics are not observed in streptozotocin–nicotinamide-induced T2DM mice fed a high-fat diet or mice fed a CDAA diet.

First-line treatment for NAFLD is lifestyle intervention to achieve weight reduction [35]. However, efficacious drugs approved for NAFLD treatment are lacking. Metformin and pioglitazone are insulin-sensitizing agents. In a meta-analysis, metformin with lifestyle intervention did not improve aminotransferase levels and liver histology compared with lifestyle intervention alone [36]. Pioglitazone has beneficial effects on NASH, but there are problems in terms of long-term efficiency and safety, such as association with the side effects of cardiovascular disease, congestive heart failure, bladder cancer, and bone loss [37, 38].

SGLT2Is have been developed for T2DM treatment but are expected to have therapeutic effects on NASH. Given the mechanism of action of SGLT2Is, urinary tract and genital infections are considered as side effects. However, Vivian et al. [18] reported that no safety concerns were identified in a 12-week treatment study. SGLT2Is have been shown to decrease aminotransferase levels in T2DM patients [39]. Ipragliflozin has been demonstrated to improve liver steatosis in T2DM mice [21]. However, whether SGLT2Is have potential effects on NASH are not known. Therefore, we investigated the effect of ipragliflozin on NASH in AMLN mice.

**Fig 5:**
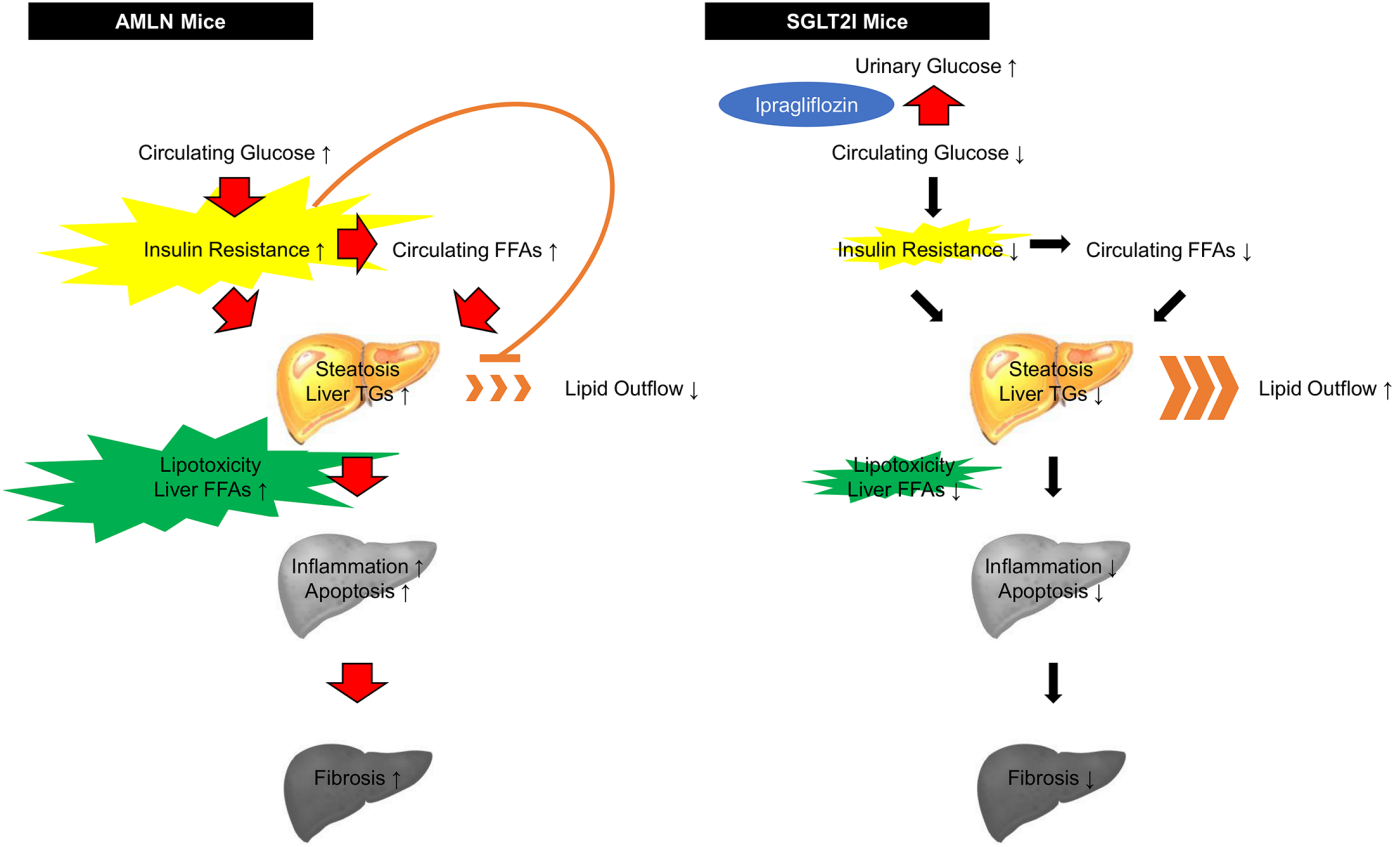
Mechanism of the effect of ipragliflozin on NASH in AMLN and SGLT2I mice (schematic). TGs, triglycerides; FFAs, free fatty acids.

A scheme illustrating the effects of ipragliflozin on NASH in our study is shown in Fig 5. Ipragliflozin improves hyperglycemia by inducing urinary excretion of glucose. The ipragliflozin dose dependently increases urinary glucose excretion in various types of mice such as normal and streptozotocin–nicotinamide-induced diabetic mice [20–23]. In this study, we considered that ipragliflozin improved insulin resistance by increasing urinary glucose excretion.

Insulin resistance is a key factor affecting liver steatosis [40, 41]. In the insulin-resistant state, serum concentrations of FFAs are elevated [42] and have an important role in liver steatosis [43]. In the present study, ipragliflozin ameliorated liver steatosis by improving insulin resistance and decreasing FFA levels (Tables 1, 2 and Fig 1).

It is thought that the pathogenesis of NASH involves lipotoxicity, gut/nutrient-derived signals, adipocytokines, and genetic factors [44]. Lipotoxicity is excess accumulation of lipids in non-adipose tissues and is caused by an increase in the flux of FFAs within hepatocytes [45, 46]. Previous reports showed that lipotoxicity leads to cellular injury and death and that this is one of the key factors of inflammation and fibrosis [47–49]. Recent studies have suggested that hepatocyte apoptosis induced by FFAs is important in the pathogenesis of NASH [50]. Previously, we reported that a reduction in β-oxidation, and the blockade of export of very-low-density lipoprotein, are key factors in the pathogenesis of NASH [51], and therefore, this report is considered to confirm the theory of lipotoxicity. In the present study, ipragliflozin improved the lobular inflammation score and the number of apoptotic cells by reducing hepatic levels of FFAs (Table 2, Figs 1 and 2). In SGLT2I mice, β-oxidation and export of very-low-density lipoprotein were accelerated by upregulation of expression of PPARα, CPT1A, and MTTP genes (Fig 3). It is thought that these genes are negatively regulated if systemic inflammation (e.g., insulin resistance) is present [52–54]. SGLT2I mice showed increased outflow of lipids from the liver because ipragliflozin improved insulin resistance and liver inflammation. Hence, we hypothesize that ipragliflozin improved insulin resistance and lipotoxicity and as a result, it decreased liver inflammation and hepatocyte apoptosis. Liver fibrosis is a marker for the progression of liver disease. Ipragliflozin decreased areas of SR staining and α-SMA staining, and lowered mRNA levels of collagen 1α1 and α-SMA (Fig 3). Our results suggest that ipragliflozin improves insulin resistance and indirectly affects the pathogenesis of NASH.

Hayashizaki-Someya et al. reported that ipragliflozin has a prophylactic effect on hepatic steatosis and fibrosis in rats fed a CDAA diet [24]. Although CDAA-diet rat model is a nutrient deficiency model and there are few different clinical conditions between that model and human NASH, ipragliflozin suppressed both hepatic steatosis and fibrosis. Ipragliflozin may directly affect NASH pathogenesis regardless of improvement of insulin resistance, when we consider this results.

Obesity is strongly associated with NAFLD. In the obese population, it has been reported that >74% have NAFLD [9]. Other reports have shown that ipragliflozin decreases body weight and the weight of epididymal adipose tissue in mice [21, 23]. In T2DM patients, a selective inhibitor of SGLT2, dapagliflozin, has been shown to reduce body weight [55]. Yokono et al. [23] reported that the mean daily intake of food was slightly greater in ipragliflozin-treated rats fed a high-fat diet. In the present study, although there was no difference in daily food intake, it was slightly greater than AMLN mice in SGLT2I mice. Ipragliflozin decreased liver weight significantly, but body weight and weight of epididymal adipose tissue were comparable (Table 1). We hypothesize that this discrepancy occurred due to differences in food intake, protocols or strains of mice. Although there was no difference in body weight, it is a surprising result that in SGLT2I mice, the clinical and pathological conditions were improved in comparison with the AMLN mice.

This study had three main limitations. First, we used just one dose of ipragliflozin. D14101901 was the AMLN diet with 40 mg ipragliflozin L-proline/kg of diet. This dose is 3 mg/kg/day and similar to a clinical dose of 50 mg. Second, we administered ipragliflozin only, so anti-DM drugs could not be compared with ipragliflozin. Third, although we fed C57BL/6J mice with an AMLN diet for 20 weeks, only one AMLN mouse showed hepatocyte ballooning.

In summary, we showed that ipragliflozin improved the pathogenesis of NASH by improving insulin resistance and lipotoxicity in NASH-model mice with T2DM. These results suggest a therapeutic effect of ipragliflozin on NASH with T2DM.

## Supporting Information

S1 Fig The experimental protocol. (DOCX)

S1 Table Scoring system and fibrosis stage. (DOCX)

## Abbreviations

NAFLD: nonalcoholic fatty liver disease
NAFL: nonalcoholic fatty liver
NASH: nonalcoholic steatohepatitis
T2DM: type 2 diabetes mellitus
SGLT2I: sodium glucose cotransporter 2 inhibitor
Hb: hemoglobin
CDAA: choline-deficient L-amino acid-defined
AMLN: Amylin liver NASH model
BD: basal diet
AST: aspartate aminotransferase
ALT: alanine aminotransferase
FFA: free fatty acid
HOMA-IR: homeostasis model assessment of insulin resistance
TG: triglycerides
H&E: hematoxylin and eosin
MT: Masson’s trichrome
SR: Sirius red
α-SMA: α-smooth muscle actin
PCR: polymerase chain reaction
mRNA: messenger RNA
ACC: acetyl-CoA carboxylase
SREBP1c: sterol regulatory element-binding protein 1c
PPARα: peroxisome proliferator activated receptor α
CPT1A: carnitine palmitoyltransferase 1A
MTTP: microsomal triglyceride transfer protein
