# Supplementary material for: The Selective SGLT2 Inhibitor Ipragliflozin Has a Therapeutic Effect on Nonalcoholic Steatohepatitis in Mice
Source: PLoS One. 2016 Jan 5;11(1):e0146337. doi: 10.1371/journal.pone.0146337 (PMC4701474; doi:10.1371/journal.pone.0146337)
Supplement: S1 Fig — (DOCX) [file pone.0146337.s001.docx]

**S1 Fig.**

**
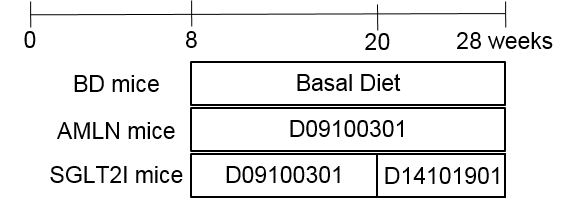
**

**The experimental protocol.**

BD mice were fed basal diet for 20 weeks. AMLN mice were fed D09100301, Amylin liver NASH model (AMLN) diet, for 20 weeks. SGLT2I mice were fed D09100301 for 12 weeks and D14101901, AMLN diet with 40 mg ipragliflozin L-proline/kg of diet, for 8 weeks.
