# Supplementary material for: The Selective SGLT2 Inhibitor Ipragliflozin Has a Therapeutic Effect on Nonalcoholic Steatohepatitis in Mice
Source: PLoS One. 2016 Jan 5;11(1):e0146337. doi: 10.1371/journal.pone.0146337 (PMC4701474; doi:10.1371/journal.pone.0146337)
Supplement: S1 Table — (DOCX) [file pone.0146337.s002.docx]

**S1 Table.**

**Scoring system and fibrosis stage.**

| **NAFLD Activity Score** | | |
| --- | --- | --- |
| Item | Definition | Grade |
| Steatosis | <5% | 0 |
|  | 5–33% | 1 |
|  | 33–66% | 2 |
|  | >66% | 3 |
| Lobular Inflammation | None | 0 |
|  | <2 foci/field | 1 |
|  | 2–4 foci/field | 2 |
|  | >5 foci/field | 3 |
| Hepatocyte Ballooning | None | 0 |
|  | Few ballooned cells | 1 |
|  | Many ballooned cells | 2 |
| **Fibrosis stage** | | |
| Item | Definition | Stage |
| Fibrosis stage | Isolated perisinusoidal or portal/periportal fibrosis | 1 |
|  | Perisinusoidal and portal/periportal fibrosis | 2 |
|  | Bridging fibrosis | 3 |
|  | Cirrhosis | 4 |

Items, definitions, grades, and stages used in this study.
